# New developments in our understanding of ankylosing spondylitis pathogenesis

**DOI:** 10.1111/imm.13242

**Published:** 2020-08-17

**Authors:** Aniruddha Voruganti, Paul Bowness

**Affiliations:** ^1^ Exeter College University of Oxford Oxford UK; ^2^ Nuffield Department of Orthopaedics Rheumatology and Musculoskeletal Science (NDORMS) Botnar Research Centre University of Oxford Headington Oxford UK

**Keywords:** ankylosing spondylitis, immunometabolism, microbiome, spondyloarthritis

## Abstract

Ankylosing spondylitis (AS) is a common immune‐mediated inflammatory arthritis with a strong genetic predisposition. We review recent data from genetic and animal studies highlighting the importance of Type 17 immune responses. Furthermore, the efficacy (or lack thereof) of different anti‐cytokine monoclonal antibodies has highlighted the diversity of Type 17 immune cells and cytokines critical to AS and related spondyloarthritis pathogenesis. Recent studies have strongly implicated the gut microbiome in AS. Finally, we propose that the local metabolic environment of the joint may have a key role in driving AS, and present a novel model of AS pathogenesis.

## Graphical abstract

Drawing on recent evidence from animal models and human trials in AS and related diseases, we propose a multifactorial model for AS pathogenesis. This involves an interplay between the immune system (particularly the Type 17 immune response), the gut microbiome and the local metabolic environment within the gut and joints. Key aspects of the model include a high degree of redundancy, and plasticity in the pathogenetic pathways of AS.

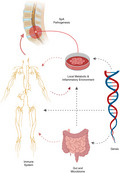

**Abbreviations**
APC: antigen‐presenting cell
AS: ankylosing spondylitis
ASAS: assessment in ankylosing spondylitis
AxSpA: axial spondyloarthritis
BASDAI: Bath AS Disease Activity Index
ER: endoplasmic reticulum
ERAP: endoplasmic reticulum aminopeptidase
FOXP3: forkhead box P3
G‐CSF: granulocyte colony‐stimulating factor
GM‐CSF: granulocyte‐macrophage colony‐stimulating factor
GWAS: genome‐wide association studies
KIR3DL2: killer cell immunoglobulin‐like receptor 3DL2
HSD: high‐salt diet
HLA‐B27: human leucocyte antigen B27
IBD: inflammatory bowel disease
IL: interleukin
IL‐23R: interleukin 23 receptor
IMID: immune‐mediated inflammatory disease
MRI: magnetic resonance imaging
NICE: National Institute for Health and Care Excellence
NSAIDs: non‐steroidal anti‐inflammatory drugs
SGK1: serum and glucocorticoid‐regulated kinase 1
SpA: spondyloarthritis
RANKL: receptor activator of nuclear factor kappa‐Β ligand
RORγt: RAR‐related orphan receptor gamma
TGF‐β3: transforming growth factor beta 3
TH17: type 17 helper T‐cell
TREG: regulatory T‐cell
TNF: tumour necrosis factor
TNFi: TNF inhibition

## Introduction

Ankylosing spondylitis (AS) is an immune‐mediated inflammatory arthritis that is part of a larger class of spondyloarthropathies (SpA), which includes reactive arthritis, enteropathic arthritis and psoriatic arthritis. AS mainly affects the axial skeleton, particularly the sacroiliac and spinal joints, resulting in severe chronic pain and disability. In advanced disease, AS is characterized by ankylosis – which is the formation of new bone – resulting in the fusion of vertebrae, reduced mobility and long‐term disability. AS usually presents first between the ages of 20 and 30 years with back pain and stiffness. Complications include iritis, an increased risk of osteoporosis and spinal compression fractures, and cardiovascular disease. A diagnosis of AS has traditionally been confirmed with sacroiliac joint X‐rays satisfying the Modified New York Criteria.^1^, ^2^ More recently, the international ASAS (ASsessment in Ankylosing Spondylitis) group has defined criteria for axial spondyloarthritis (AxSpA) that are not reliant on X‐ray, give more weight to magnetic resonance imaging (MRI) and HLA‐B27 status, and have been accepted for treatment decisions by the UK National Institute for Health and Care Excellence (NICE).^3^, ^4^ Here, we have used the terms AS and AxSpA relatively interchangeably, although noting that these are distinct diseases, and that there may be subtle differences in the pathogenesis of AxSpA in the context of psoriasis or inflammatory bowel disease (IBD). Importantly, the move away from radiography towards MRI (using the new Assessment of SpondyloArthritis International Society criteria) has allowed us to diagnose this disease before it progresses to its ankylosing form, and to initiate treatment earlier with better long‐term outcomes.

**Figure 1:**
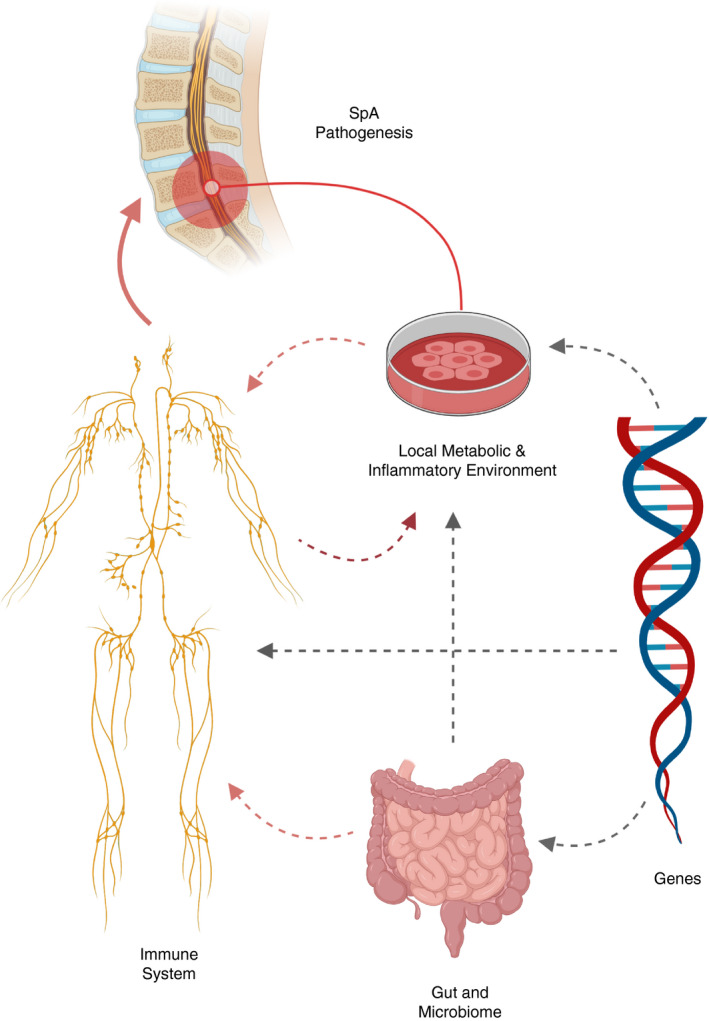
Key factors in ankylosing spondylitis (AS) pathogenesis.

Whilst treatments are available to alleviate the symptoms of AS, there is currently no cure. Physiotherapy is a mainstay of treatment, along with pain management with non‐steroidal anti‐inflammatory drugs (NSAIDs) and analgesia.^4^, ^5^ Advances in our understanding of the pathogenesis of AS have led to new disease‐modifying drugs that target pathways of pathogenesis, discussed later. The currently licensed and NICE‐approved drugs in the UK are anti‐tumour necrosis factor (TNF) therapies and the anti‐interleukin (IL)‐17 monoclonal antibody secukinumab (Fig. 1).

This review will discuss the multifactorial pathogenesis of AS. We will review evidence from recent therapeutic trials highlighting the heterogeneity of the Type 17 immune response, together with insights into the role of the gut microbiome and immunometabolomics. We propose a disease model involving complex interplay between the immune system, the gut and the local immunometabolic environment of the joint, under the influence of a clear genetic basis.

## Genetic studies in AS confirm high heritability and identify multiple immune‐related genetic loci in pathogenesis

The risk of siblings or first‐degree relatives of AS patients having AS is higher than that of the general population, and there is a high degree of concordance in twins. Such studies have led to heritability calculations of approximately 90% for AS.^6^ The strongest genetic association with AS is the HLA‐B*27 gene.^7^ The prevalence of AS in a population depends on the HLA‐B*27 prevalence in that population.^8^ The amino acid in position 97 of the HLA‐B gene is hypothesized to be implicated in the functional differences in these allelic variants.^9^ Several theories have been put forward to explain the pathogenic mechanisms of the HLA‐B27 association with AS.^10^ Altered peptide binding may affect the risk of autoimmunity by changing the likelihood of presenting an autoimmunity‐inducing peptide to cytotoxic CD8^+^ T‐cells.^11^, ^12^ Other theories suggest that unconventional HLA‐B*27 variants homodimerize instead of heterodimerize, and increase IL‐23 production by triggering the unfolded protein response of the endoplasmic reticulum (ER).^13^ These HLA homodimers and open conformers can also be expressed on the surface of various antigen‐presenting cells (APCs), and bind to various immunoreceptors, including killer cell immunoglobulin‐like receptor 3DL2 (KIR3DL2) expressed on T‐ and natural killer (NK) cells,^14^ and leucocyte immunoglobulin‐like receptors (LILR) on monocytes and B‐cells.^15^ These open conformers can thus have broadly immunostimulatory and T_H_17‐stimulating effects.^16^

Genome‐wide association studies (GWAS) have linked multiple genetic loci to the pathogenesis of AS, with 48 identified by Cortes and colleagues,^17^ and over 100 identified by cross‐disease comparison.^18^ Interestingly, only about a quarter of the heritability of AS is currently accounted for by the genetic loci linked to the disease – of which 20·1% is linked to HLA‐B*27 alone,^12^, ^17^ with IL‐23R and endoplasmic reticulum aminopeptidase (ERAP)1 being the next most potent, but far weaker, genetic factors.^19^ Nevertheless, these have recently come to the fore as potentially important mechanistic actors for the disease. IL‐23R involvement implicates T_H_17 responses (see next section), and ERAP1 strongly implicates antigen presentation.^17^, ^20^ ERAP1 is an aminopeptidase whose effect is epistatically linked to HLA‐B*27, i.e. its effects are only seen in individuals who are HLA‐B27‐positive. ERAP2 is also genetically linked to AS. These aminopeptidases are molecular rulers that trim peptides to a specific length before they bind to the HLA molecule – changes in their activities could result in arthritogenic peptide formation, loss of protective peptides or altered HLA‐B27 folding and/or trafficking (see above). Additional HLA allotypes and genes within the HLA region have been linked to AS, both protective and pathogenic.^9^ Another important class of genes that has been linked to AS includes a class of glycosphingolipid sensors encoded by the *GPR* genes – including *GPR25*, *GPR35* and in particularly *GPR65*, discussed later.

Finally, despite not having a large GWAS footprint, some pathways involving multiple genes such as *TNF* have functional importance due to their role in generalized inflammation. Indeed, TNF inhibition (TNFi) is a mainstay of AS treatment.^2^, ^21^, ^22^, ^23^, ^24^ However, whilst TNFi efficacy is generally dose‐dependent, TNF inhibition does not work in all patients and there is heterogeneity in treatment efficacy.^25^ We propose that there is thus significant scope for subtyping SpA based upon genetics and/or immunological phenotype (see below), and trialling stratified treatments.

Lastly, it is important to note that recent studies have identified a significant overlap in genes associated with AS with those in IBD, psoriasis, psoriatic arthritis and anterior uveitis.^12^ This likely drives the significant clinical and pathogenic overlap seen between these conditions, with evidence suggesting shared dysregulation immune responses at mucosal sites and heightened type 17 inflammation (see below).

## Type 17 immunity is heightened in AS, type 17 family responses are heterogeneous and exhibit plasticity

Our current understanding suggests that the IL‐17‐IL‐23 axis and more broadly Type 17 immune responses are of particular relevance to the pathogenesis of AS. An overactive IL‐17‐IL‐23 pathway can explain both the key inflammatory aspects of disease (including enthesitis) and the paradoxical feature of systemic bone loss coupled with pathological bone neogenesis.^26^ Evidence for the involvement of this axis in pathology will be considered after a brief mechanistic overview of its physiology.

IL‐23 is a heterodimeric cytokine, secreted by myeloid cells including monocytes, macrophages and dendritic cells, often in response to danger‐associated molecular patterns. IL‐23 is crucial for the differentiation of T helper cells into the T_H_17 phenotype, polarizing and stabilizing this pro‐inflammatory phenotype.^27^ IL‐17A is a proinflammatory cytokine that is also involved in maintaining mucosal immunity and barrier function, particularly in the skin and gut. It is produced by a variety of cell types, including CD4 (T_H_17) cells, CD8^+^ and γδ T‐cells and innate lymphoid cells.^28^ Al‐Mossawi *et al*. also highlighted a possible role for granulocyte‐macrophage colony‐stimulating factor (GM‐CSF) production in AS. GM‐CSF is produced both by and independent of T_H_17 T‐cells, and is increased in synovial T‐cells and innate lymphoid cells.^28^ We propose that SpA pathogenesis may be characterized by T_H_17 polyfunctionality (i.e. production of multiple cytokines) together with T‐cell plasticity (with change of phenotype after entry into joints and entheses). Evidence for T helper cell plasticity cells has been demonstrated in juvenile inflammatory arthritis and in mouse models of arthritis.^29^, ^30^ Thus, T‐cells including T_REG_ cells can under certain circumstances become proinflammatory, and this process might contribute to SpA pathology.

## Animal models of SpA support roles for HLA‐B27, the gut microbiome and type 17 immunity

Whilst no animal models recapitulate human SpA, several have offered important insights into pathogenic processes. The HLA‐B27 transgenic rat model has shown the critical role of HLA‐B27, gut flora and bone marrow‐derived leucocytes (including T_H_17 cells) in pathogenesis, whilst arguing against a classical role of CD8^+^ T‐cells in disease.^31^, ^32^ Of interest is that HLA‐B27 homodimers have been demonstrated in both human SpA and HLA‐B27 rat tissue.^33^

Studies using mouse models have shown a key role for IL‐23 in activating T‐cells (including γδ T‐cells) in entheses, and in inducing expression of IL‐17A and other proinflammatory cytokines such as IL‐22 and IL‐17F.^34^, ^35^ Further support for the role of the T_H_17 axis comes from DBA/1 mice, which spontaneously develop AS‐like enthesitis but do not do so when IL‐17A is neutralized with antibodies.^36^ Furthermore, male BXSB × NZB (which also spontaneously develop ankylosing enthesitis when caged in groups) have shown to have accumulated IL‐17 producing T_H_17 cells.^37^

Gaublomme and colleagues used single‐cell RNA sequencing to demonstrate heterogeneity within the T_H_17 pool of cells.^38^ This has important implications for treatment because one can target the pathogenic subtypes of T_H_17 cells whilst preserving the beneficial mucosal immune functions of the T_H_17 axis. It was found that *GPR65* is necessary for T_H_17 pathogenicity [*GPR65* knockout mice did not develop experimental autoimmune encephalomyelitis (EAE) and did not produce IL‐17A^+^ cells when activated by IL‐23]. *GPR65* is also upregulated in those T_H_17 cells that are induced to become pathogenic (by cytokines such as IL‐1B, IL‐6 and IL‐23). *GPR65* therefore appears to be of particular importance and may be useful as a drug target.

There is also evidence to suggest that transforming growth factor beta 3 (TGF‐β3) is important in the induction of pathogenic T_H_17 cells. TGF‐β3 is dependent on IL‐23 signalling – and the *in vivo* deletion of TGF‐β3 resulted in the loss of IL‐17^+^ cells. Furthermore, T_H_17 cells induced in the absence of TGF‐β3 are significantly less pathogenic and do not cause disease when adoptively transferred to EAE mice, as compared with T_H_17 cells induced in the presence of TGF‐β3.^39^ Thus TGF‐β3 could also be a relevant target in the treatment of AS.

These studies highlight the heterogeneity of Type 17 immune responses, and the fact that pathogenicity may relate to production of additional cytokines and/or co‐expression of additional functional molecules and receptors.

## Sex differences in AxSpA and AS – to what extent do these represent genuine differences in male and female immunology and physiology?

Unusually for an immune‐mediated inflammatory disease (IMID), AS has traditionally been diagnosed more commonly in men than in women. Whilst there may be sex‐specific differences in AS that are relevant to disease pathogenesis and treatment (see below and Refs [40, 41]), in recent studies the incidence of AxSpA in women approaches that in males, perhaps due to improvements in diagnosis (early MRIs) and awareness of the condition.^42^ Thus, it is possible that AS has hitherto simply been under‐recognized in women, perhaps due to the non‐specific nature of AS symptoms and the underdiagnosis of women’s pain. Women have also been underrepresented in studies of AS, and tend to have a higher disease burden due to late diagnosis and ineffectiveness of current treatments. Nevertheless, recent immunological studies have identified clear differences between males and females, including differences in T_H_17 number. We propose that the enhanced T_H_17 responses found in males may drive the higher male prevalence of certain Type 17‐driven IMIDs, including AS and UC, compared with classical autoimmune IMIDs.^43^, ^44^ Furthermore, women also tend to have less new bone formation, making diagnosis of AS more difficult by radiography.^41^, ^45^ Although further careful epidemiological and experimental work is needed, our interpretation of currently available data is that the prevalence of sacroiliitis and AxSpA are relatively equal in males and females, perhaps with a small male bias. By contrast, radiographic AS has a more marked male preponderance.

## Evidence from human studies and therapeutic trials

Patients with AS have been found to have increased levels of circulating IL‐17 and IL‐23 compared with healthy controls.^46^ Furthermore, patients with AS also have increased numbers of pathogenic T_H_17, T_H_22 cells and IL‐17‐secreting γδ cells in their peripheral blood and accumulating in their joints.^47^, ^48^, ^49^ From a translational angle, the monoclonal antibody secukinumab, which targets IL‐17A, is effective in treating AS symptoms in clinical trials (MEASURE 1 and MEASURE 2),^50^, ^51^ and is now NICE‐approved for treatment in the UK.^52^ The Bath AS Disease Activity Index (BASDAI) score of AS patients also correlates with their serum levels of IL‐17.^53^ IL‐17 also upregulates the production of receptor activator of nuclear factor kappa‐B ligand (RANKL) and is a potent osteoclastogen in joints.^54^

IL‐17 synergizes with TNF to induce secretion of GM‐CSF^55^ and granulocyte colony‐stimulating factor (G‐CSF).^56^ GM‐CSF is thought to dysregulate haematopoietic stem cell activity in the context of experimental SpA,^57^ and the pathogenicity of T_H_17 cells was dependent on GM‐CSF in a murine model of multiple sclerosis.^58^ Al‐Mossawi *et al*.^28^ showed that patients with SpA had increased levels of IL‐17A^+^ GM‐CSF^+^ double‐positive CD4, CD8, γδ and NK cells in the blood and the joints. GM‐CSF thus represents an important future avenue for therapy. Indeed, the GM‐CSF‐blocking antibody namilumab is currently being trialled in the NAMASTE trial, a phase II double‐blind trial with 42 patients with refractory SpA.^59^

With regards to IL‐23, an open‐label study suggested that ustekinumab (which targets the IL‐23 p40 subunit, also part of IL‐12) was useful for the treatment of AS,^60^ although in subsequent trials ustekinumab did not achieve the primary end points in AS patients with TNFi failure,^61^ and has not been approved for use in AS. More recently, a randomized controlled clinical trial performed in a 2018 study showed that risankizumab, which targets the p19 subunit of IL‐23, does not show efficacy in the treatment of AS.^62^ This result was somewhat surprising, and may relate to the stage of disease or to different roles of different ‘T_H_17 family’ cytokines at different sites of inflammation.^63^ Of likely relevance is the recent description that human entheseal γδ T‐cells lacking IL‐23R can produce IL‐17.^64^

Whilst the above data correlate the IL‐23‐IL‐17 axis with disease progression, the mechanistic basis for how this pathway confers the uniquely paradoxical AS bone phenotype of increased systemic osteopaenia (and thus an increased fracture risk), coupled with bone neogenesis and ankylosis is poorly understood. It is thought that systemically, IL‐23 and IL‐17 act to promote central inflammation and the osteoclastogenesis that ensues.^27^ Locally, entheseal bone formation is thought to be due to the interaction between the pro‐enthesitic and pro‐inflammatory effects of this pathway and mechanical stress.^65^, ^66^, ^67^

Small molecule inhibitors targeting intercellular proinflammatory pathways, including those downstream of cytokine signalling, have been trialled in AS. These include the PDE4 inhibitor apremilast.^68^ PDE4 is a phosphodiesterase involved in the production of inflammatory cytokines through AMP production. Apremilast did not meet its primary end‐point in a phase 2 trial in AS,^68^ although has proved effective in PsA.^69^

Efficacy in AS was recently demonstrated for the small molecule JAK1 inhibitor filgotinib.^70^ Interestingly, neither T_H_17 nor the TNF axis have been thought to directly involve the JAK1 pathway,^71^, ^72^ although both pharmacological and siRNA inhibition of multiple JAKs and TYK2 have been shown to inhibit T_H_17 responses *ex vivo*.^73^ Tyk2 inhibition has been effective in murine SpA,^74^ and tofacitinib showed efficacy in a dose‐ranging phase 2 study in AS.^75^ Thus, similar to TNF inhibition, JAK inhibition may serve to break one of the proinflammatory cycles perpetrated within the joints of patients with SpA.

## The gut microbiome and AS

Several lines of evidence link the gastrointestinal tract with development of SpA. Thus, there is a strong association between IBD and AS, which have a high degree of co‐familiality. First‐degree relatives of AS patients are three times more likely to have IBD, and up to 70% of AS patients have subclinical gut inflammation on endoscopic and/or histological examination.^76^ This is unsurprising as the Type 17 immune response is important in maintaining mucosal barrier function, and AS patients are known to have elevated gut IL‐23 levels.^77^ Whilst AS, unlike the related SpA reactive arthritis, does not appear to be directly triggered by an infectious agent, HLA‐B*27 transgenic rats do not develop arthritis when reared under germ‐free conditions. Furthermore, patients with AS have recently been shown to have a different microbiome composition to controls, with evidence of dysbiosis.^78^, ^79^, ^80^ It should be noted that different studies using different methodologies have found somewhat differing results, with no single species or genus of bacteria being consistently found enriched (or absent) in all studies. We would hypothesize that AS may be associated with broad change in the microbiota, perhaps leading to altered Type 17 immunogenicity or altered metabolic signalling (see below) or both. There is good evidence, albeit predominantly in mice, for an interplay between gut microbiota and differing Type 17 immune responses.^81^, ^82^ Lastly, the presence or absence of HLA‐B27, in and of itself, has recently been reported to have an influence on the gut microbiome.^83^, ^84^

## The gut and joint metabolic environment and AS

Tying the genetic evidence together with new data showing alterations in gut microbiome is the concept that dysregulated immune responses may result from changes in the local immunometabolic environment. This likely occurs both in the joint and in the gut, with the latter potentially driven by gut dysbiosis.

Thus, there is emerging evidence that Type 17 immune responses are sensitive to local metabolic cues, such as salt and pH. The salt‐sensing channel Serine/Threonine protein kinase SGK1 has been shown to affect T_H_17 responses. Wu *et al*.^85^ found that SGK1 is an important downstream mediator of IL‐23. Importantly, they found that SGK1 is itself further upregulated in T_H_17 cells in response to IL‐23, stabilizing their proinflammatory phenotype. The same paper also showed that 40 mm NaCl upregulated SGK1 expression in naïve CD4 T‐cells, even in the absence of any other potentiating cytokines. In the presence of these cytokines, NaCl significantly resulted in the upregulation of IL‐17 and IL‐23 compared with control. This effect was not found in *SGK1* ^−/−^ cells, implicating SGK1 in potentiating this polarized inflammatory state. Further linking the metabolic state of the body and the gut to autoimmune arthritis, mice fed a high‐salt diet (HSD) had significantly higher T_H_17 cells in their gut lamina propria. In a subsequent study, eight male volunteers fed a HSD showed an increase in peripheral blood T_H_17 cells and a concomitant loss of gut commensal lactobacillus species.^86^ Wild‐type mice fed a HSD also developed more severe EAE compared with mice fed a normal diet, and this increased severity was abrogated in *SGK1*‐deficient mice.^85^ The question now arises as to whether a HSD could influence the instability of forkhead box P3 (FOXP3) cells in the lamina propria, as it has been found that there is a reciprocal relationship between T_H_17 cells and T_REG_ cells in the gut of autoimmune disease models.^87^, ^88^ Importantly, Wei *et al*.^89^ found that a HSD not only promoted the differentiation of IL‐17 secreting cells in the gut lamina propria, but also repressed T_REG_ cells. Interestingly, reciprocal effects of bile acid metabolites on T_H_17 and T_REG_ have recently been described in mice.^90^

The *GPR65* gene, an important GWAS locus predisposing to AS, codes for a proton sensor present on T_H_17 cells, and thus one could speculate about the association between dysregulated acid sensing of the metabolic environment and the induction of a pro‐inflammatory phenotype. Al‐Mossawi *et al*. also found that the pathogenic cells in the joints of SpA patients also had elevated expression of *GPR65*. Additionally, culturing T‐cells in an acidic environment has been shown to increase their production of GM‐CSF. It was found that blocking GPR65 reduces GM‐CSF levels of primary CD4 T‐cells; thus, GPR65 and GM‐CSF represent promising future therapeutic targets.^28^, ^57^, ^91^ Nistala *et al*.^92^ showed that IL‐17 producing cells were present in greater numbers in the joints of children with severe juvenile idiopathic arthritis, with a reciprocal relationship between the number of IL‐17 cells and T_REG_ cells. Given that T_REG_ cells can be converted to a T_H_17 phenotype by metabolic cues (including 2‐hydroyglutarate),^93^ we propose that such processes may occur in SpA and that this area warrants further investigation.

In summary, whilst there are clear mechanistic data in animal models for immunometabolic processes in driving Type 17 immunity, we should point out that evidence for immunometabolic disturbance in AS is preliminary and circumstantial. The roles of gut microbiome and the exact metabolic and ionic nature of the gut mucosa, joints and entheses are thus important questions for future research.

## Future directions, conclusions and perspectives

One of the key conclusions to be drawn from the above evidence is that T‐cell differentiation and pathogenicity are not determined by a few specified subtypes, but rather several types of T‐cells may take on a pathogenic phenotype depending on the local environment of the joint. This has important implications, because targeting a single pathway may not be very effective; the plasticity of T‐cells and dependence on non‐specific factors such as pH and Na^+^ levels results in an increase in redundancy in pathogenic pathways. Thus, plasticity and the lack of specificity confer greater redundancy to the system. Furthermore, it may be ineffective to use therapies involving T_REG_ cells to modulate disease as these may ‘defect’ to a pathogenic phenotype and potentially exacerbate disease. However, the implication is also that the targets of new effective therapies could be more ‘general actors’, as TNF has been, and the JAKs could be. This would result in a general reduction of inflammation, which breaks down positive pathogenetic cycles. More speculatively, being able to control the local metabolic environment may allow us to control the disease better. Changes in diet and lifestyle can target the gut‐immune axis; this also is supported by the idea that salt intake may control T‐cell pathogenicity. Future avenues of therapy involve targeting candidates such as GM‐CSF (the NAMASTE trial), GPR65 (in clinical trials) and SGK1 (US patent application filed).

It will also be important to understand the different subsets of T_REG_ cells, and what factors influence FOXP3 instability. It is indeed possible that SGK1 and GPR65 are linked to (the loss of) FOXP3 expression. Future research could involve studying synovial fluid from AS patients, and understanding its immunometabolic properties and whether this affects T‐cell plasticity in the joint.

**Figure 2:**
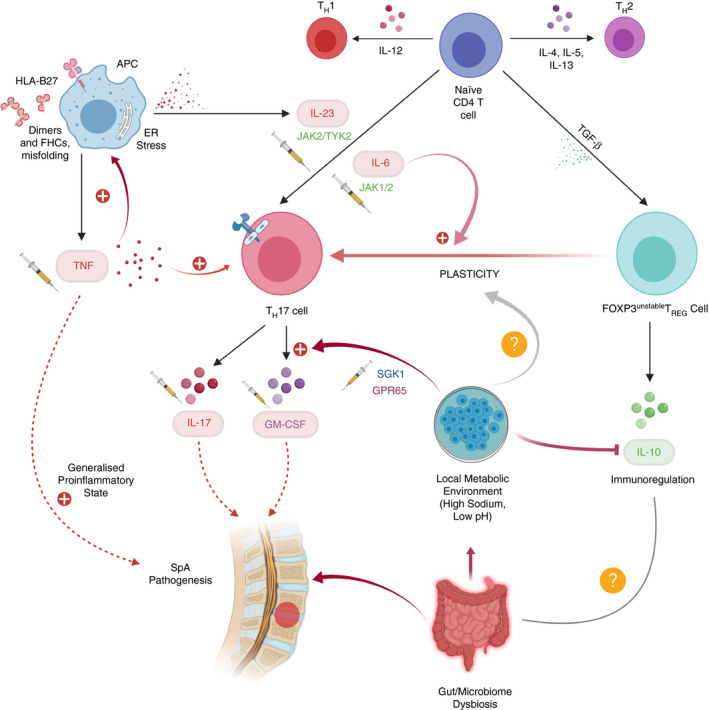
Model showing interplay of factors affecting CD4^+^ T‐cell differentiation and their likely roles in ankylosing spondylitis (AS) pathogenesis. We include speculation about links between the metabolic environment and T‐cell plasticity. Existing or potential drug targets are indicated with a syringe symbol next to them.

The diagram below details the variety of factors that are at play in the pathogenesis of AS (Fig. 2).

In conclusion, AS is a disease that is mediated by multiple cell types and pathogenic pathways. Recent research has highlighted the importance in disease pathogenesis of the gut‐immune axis, the local immunometabolic environment and T‐cell plasticity – particularly a drive to heightened and likely aberrant Type 17 immunity.

## Disclosures

AV declares that he has no conflict of interest.

## Data Availability

Data sharing not applicable to this article as no datasets were generated or analysed during the current study.
